# Hypertensive Patients' Knowledge of Risk Factors and Warning Signs of Stroke at Felege Hiwot Referral Hospital, Northwest Ethiopia: A Cross-Sectional Study

**DOI:** 10.1155/2019/8570428

**Published:** 2019-06-23

**Authors:** Addisu Taye Abate, Netsanet Bayu, Tesfamichael G. Mariam

**Affiliations:** University of Gondar, College of Medicine and Health Sciences, School of Nursing, Department of Medical Nursing, Gondar, Ethiopia

## Abstract

**Background:**

Stroke is a neurological condition which is a major cause of death and disability in many low- and middle-income countries. However, several modifiable risk factors are becoming significant. Hypertension is the most common stroke risk factor globally as well as in our country, Ethiopia.

**Objective:**

The aim of this study was to assess hypertensive patients' knowledge of risk factors and warning signs of stroke at Felege Hiwot Referral Hospital, Northwest Ethiopia, 2018.

**Method:**

An institutional based cross-sectional study design was conducted in May 01–30/2018. A total of 278 hypertensive patients were enrolled and systemic random sampling technique was employed to select the study participants. Data was collected through an interviewer-administered questionnaire. The collected data was entered into Epi info 7 and exported to SPSS version 22 for analysis. Binary and multivariable logistic regressions were used and* P* value ≤ 0.05 was considered as statistically significant.

**Result:**

Out of the total 284 selected hypertensive patients, 278 of them responded completely with a response rate of 97.9 %. Of these, more than three fourths, 214 (77%) and 201 (72.3%), of them did not identify any risk factors and warning signs of stroke, respectively, with an overall proportion of only 18.3% of them having good knowledge towards stroke. Risk factor of stroke most commonly known to the respondents was physical inactivity (21.58%), whereas hypertension was the least described risk factor (3.6%). Regarding stroke warning signs, sudden weakness on one side of the body (35.97%) was the most commonly known warning sign of stroke to the respondents. Being able to read and write, being young, urban residence, and having sufficient monthly income were significantly associated with the overall knowledge of hypertensive patients towards stroke. These findings suggest the need for emphasizing on stroke education regarding its risk factors and warning signs through public or social media and health education targeting to low-income high risk subjects.

## 1. Introduction

Stroke or cerebrovascular accident, which considerably affects the individuals' entire quality of life, is the third leading cause of death worldwide, with an incidence of approximately 15 million cases per year [1], and its fatality is more in Sub-Saharan Africa countries, which showed that 85% of global death from stroke occurred in these countries [2, 3]. In Ethiopia, lines of evidence showed that stroke is the commonest cause of admissions (45%) from other neurologic diseases [4] and it accounted for 28,320 (4.71%) of total deaths in Ethiopia [5].

Hypertension is described as one of the most common causes of stroke; and the incidence of stroke among hypertensive patients is increasing in developing countries [6]. Unlike that of developed countries, ischemic stroke, which is mostly associated with poor control of hypertension, is the commonest type of stroke in Africa [7–9].

Identification of the major lifestyle risk factors of stroke and its warning signs has a direct implication for the prevention of stroke with the possible therapeutic measures in high risk group such as hypertensive patients [10]. In addition, poor knowledge leads to low compliance in making use of preventive programmes [11, 12]. Despite this, most of Ethiopian hypertensive patients primarily presented to traditional healers who would not provide correct knowledge about stroke; and there is also lack of baseline data regarding knowledge of hypertensive patients towards stroke. Thus, patients are more likely to develop stroke and its complication [13, 14]. As a result, hypertensive related stroke death in Ethiopia has reached 28,320 (4.71%) of total deaths [14], which tells us how big the problem is and that it needs immediate attention. In this regard the results of this study would provide information on the knowledge of hypertensive patients towards stroke. Therefore, the aim of this study is to assess knowledge of hypertensive patients towards stroke risk factors and warning signs at Felege Hiwot Referral Hospital in Bahir Dar town, Northwest Ethiopia, 2018.

## 2. Methods 

### 2.1. Study Design, Setting, and Period

An institution based cross-sectional study was conducted on 278 patients attending follow-up on hypertension in May 01–30/2018 at Felege Hiwot Referral Hospital, which is found in Amhara national regional state, Ethiopia.

### 2.2. Sample Size Determination and Procedure

The source population were all hypertensive patients who were on follow-up at Felege Hiwot Referral Hospital and those individuals who were under follow-up during data collection time were considered as a study population. All hypertensive patients who were on follow-up visit for at least 6 months during the study period and aged above or equal to 18 years old were included, whereas patients who already developed stroke before data collection period and those who were critically ill, with severe mental illness, or who were unable to provide the required information by themselves were excluded. To determine the required sample size of 284 participants, single population proportion formula was used with the assumption of 95% confidence interval, 5% margin of error, and 50% proportion of hypertensive patients who have good knowledge towards stroke, as there was no similar study in the area. Systematic random sampling technique was utilized; sampling interval “K” value was calculated as K = N/nf, where N = the expected number of hypertensive patients per month = 814 and nf = final sample size = 284 which gives a sampling interval of three. Thus, using patients' record order which was listed in follow-up appointment as a sampling frame, participants were selected in every 3 number intervals until reaching the total sample size and the first participant was selected by lottery method.

### 2.3. Data Collection Method and Survey Instrument

Data collection was performed by four BSc nurses through an interviewer-administered questionnaire. The data collectors were properly trained on the instrument and ways of approaching the patients and how to obtain permission for an interview prior to the data collection process for three days. A standardized questionnaire already used in previous studies to assess knowledge of stroke risk factors and warning signs was adapted and applied for the study [13, 14]. It comprises the sociodemographic variables, stroke risk factors, and stroke warning signs. The Stroke Recognition Questionnaire (SRQ) was used to determine whether hypertensive patients could identify stroke risk factors and symptoms. It uses a list of 10 stroke symptoms, 10-non-stroke related symptoms, 10 risk factors, and 10 non-stroke risk factors of stroke. Study subjects indicate by marking “yes” or “no” whether items on the list are stroke symptoms or risk factors. Then study participants' knowledge of stroke symptoms and warning signs was categorized based on the numbers of stroke symptoms and warning signs, respectively. In this regard, individuals with good knowledge of stroke symptoms could identify 5–10 stroke symptoms. Similarly, individuals who identified 5–10 stroke warning signs were categorized as having good knowledge towards stroke warning signs [15, 16]. To determine the overall knowledge score, closed-ended questions were used. In the closed-ended questionnaire, the maximum total score was 18 points; each question accounts 1 point for correct and 0 for incorrect answers; and the overall knowledge was assessed from the total score that was scored by the patients. After all, participants with a total knowledge score of 50% and above were considered as having good knowledge [15]. The analyses in this article focused on the questions on general stroke knowledge. Initially, the questionnaire was translated from English to the official/local language of the region (Amharic); then it was retranslated to English language to ensure consistency. To test the fitness of the questionnaire for the study setting, the data collection tool was pretested on 28 adult hypertensive patients who were not included in the final analysis.

### 2.4. Operational Definition

#### 2.4.1. Body Mass Index (BMI)

It is the ratio of weight in kilogram over the height in meter square; and participants with BMI >30 kilogram per meter square were considered as obese.

#### 2.4.2. Hypertension

A diagnosis of high blood pressure: a doctor or health worker had told the participant that they had elevated blood pressure. It is defined as systolic blood pressure greater than or equal to 140 mmHg and/or diastolic blood pressure greater than or equal to 90 mmHg and/or receiving treatment for high blood pressure.

#### 2.4.3. Physical Inactivity

It refers to participants who did not get 30–60 minutes of aerobic exercise three to four times per week.

#### 2.4.4. Income

Those study participants with ≥ 5000 Ethiopian birr/month were classified as having sufficient income.

#### 2.4.5. Smoker

It refers to those participants who smoke any tobacco products (such as cigarettes, cigars, or rolled tobacco).

#### 2.4.6. Alcohol Drinker

It refers to those participants who consume a drink containing alcohol [17].

### 2.5. Statistical Analysis

All the data was checked visually, coded and entered into Epi Info version 7, and exported to Statistical Package for Social Sciences (SPSS) version 21 for analysis. Frequencies, percentages, and summary statistics like mean and standard deviation were examined to describe the data. Binary logistic regression was run to see the crude significant relations of each independent variable with the general knowledge score of hypertensive patients towards stroke. Then variables with* P* value ≤ 0.2 in bivariable logistic regression were again entered into multivariable logistic regressions. Finally, significant factors were identified based on adjusted odds ratio (AOR) included in 95% confidence level at* P* value ≤ 0.05.

### 2.6. Ethical Considerations

Ethical clearance was obtained from the Ethical Review Committee (ERC) of the School of Nursing, University of Gondar. An official letter of permission was obtained from Felege Hiwot Referral Hospital administration as a getting way. After explaining the purpose of the study, verbal informed consent was obtained from each of the study participants. Personal identifiers were not included in the written questionnaires to ensure participants' confidentiality.

## 3. Results

### 3.1. Sociodemographic Characteristics of Study Participants

Out of the total 284 selected hypertensive patients, 278 of them responded completely with a response rate of 97.9 %. As it is illustrated in Table 1, almost half, 145 (52.2%), of them were female and majority of the participants, 211 (75.9%), were aged greater than 45 with the mean age of 54.4 SD that ranged from 19 to 88. Regarding their educational status, about 154 (55.4%) of them were unable to read and write (Table 1).

### 3.2. General Knowledge of Hypertensive Patients about Stroke

Only 60 (21.6%) of hypertensive patients correctly define stroke as a condition that results from disruption of blood supply to the brain; 24 (8.6%) of the participants think stroke is the same as heart attack and a small proportion of patients, 44 (15.8%), correctly identified that stroke is a disease of the blood vessels in the brain. Based on the knowledge assessment tool, the minimum and maximum scores were 0 and 16 out of 18 with the mean score of 2.7 and only 51 (18.3%) of the participants had good knowledge towards stroke.

### 3.3. Knowledge of Risk Factors of Stroke among Hypertensive Patients

**Figure 1:**
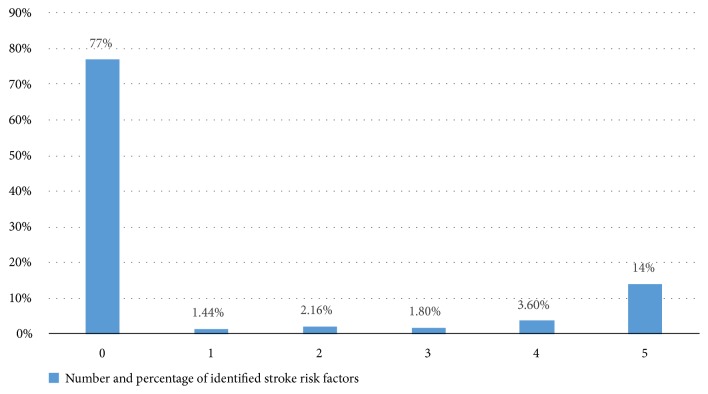
Number and percentage of identified stroke risk factors.

From the total participants, most of them, 214 (77%), did not identify any risk factors of stroke; 39 (14%) identified 5 risk factors; 10 (3.6%) identified 4 risk factors; and only 5 (1.8%) identified 3 risk factors of stroke. The stroke risk factors most commonly known to the respondents were physical inactivity (21.6%), being obese (20.1%), and drinking alcohol (18.7%). In contrast with this, hypertension was the least described risk factor, in which only 10 (3.6%) of participants reported hypertension as a risk factor of stroke (Figure 1).

### 3.4. Knowledge of Warning Signs of Stroke among Hypertensive Patients

Regarding knowledge component of major warning signs of stroke, about 217 (77.3%) of participants did not identify any warning signs of stroke. Whereas 40 (14.4%) of them identified 5 and more warning signs, 15 (5.4%) identified 4 warning signs and 2 (0.7%) identified 3 warning signs. Sudden weakness or paralysis on one side of the body (35.9%) and unusual severe headache (16.2%) were the most commonly known signs and symptoms of stroke to the respondents.

### 3.5. Factors Associated with Knowledge of Hypertensive Patients towards Stroke

In the present study, hypertensive patients able to read and write were more knowledgeable (AOR=7.128, CI 95% 2.298-22.108) than those who were unable to read and write. Regarding age, young respondents (age less than 45) were more likely to have good knowledge (AOR=2.56, CI 95% 1.115-6.015) than respondents who aged 45 and above. Similarly, urban residents were more knowledgeable (AOR=3.2, CI 95% 1.042-9.874) compared to the rural residents; and study participants with sufficient monthly income were also more likely to have good knowledge (AOR=2.756, CI 95% 1.225-6.200) than participants with insufficient monthly income (Table 2).

## 4. Discussion

This study found that most of hypertensive patients have extremely limited knowledge of stroke risk factors, are not familiar with its warning signs, and are not aware that stroke is a disease of the blood vessels in the brain. Surprisingly the respondents' knowledge regarding stroke risk factors was almost similar with that of the warning signs of stroke, in which more than three fourths (77% and 77.3%) of respondents did not identify any risk factors and warning signs of stroke, respectively. In general, only 18.3% of them had good knowledge towards stroke. This finding is contradicted with other studies in which at least half of the respondents knew one or more of the known risk factors of stroke [13, 16–18]. Likewise, in contrast with the good knowledge of stroke reported in previous studies among patients in Saudi population [19–21], only few respondents in this study (15.8%) knew stroke as a disease of the blood vessels in the brain. The possible reason for this difference might be due to the fact that half of participants in the current study were from rural communities, where education, income level, health facilities, and opportunity for education are generally expected to be lower; and these subjects are less likely to have exposure for new information including different social media.

In the current study, hypertension was the least described risk factor (3.6%). However, it has been reported as an important risk factor [22], and it is extremely lower than earlier studies which have reported higher proportions in risk factor profile of stroke patients [23, 24]. The possible difference in identification of hypertension as an important risk factor might be due to variability in health care systems regarding health education delivery; and it may be also due to the fact that majority of participants in the current study were uneducated with insufficient income who are less likely to get hypertension related information through internet, newspaper, and watching television.

The commonest stroke warning sign recognised by the patients was sudden weakness or paralysis on one side of the body (35.9%), followed by unusual severe headache (16.2%); and it is comparable to finding of Kuwaiti study, in which sudden weakness or paralysis on one side of the body was reported by 36.4% of participants [25].

The current study found a positive relationship between knowledge of stroke with hypertensive patients' age and educational level, in which those who were younger with high level of education participants had better knowledge regarding stroke risk factors and warning signs. These findings are consistent with previous studies [17, 18, 26]. This might be due to the fact that low level of education and old age may lead to limited interaction with society and therefore less interest in following medical developments, thereby resulting in a low level of health care knowledge and attitude about stroke. Moreover, financial status and education were also found to be the determinants of knowledge towards stroke [27]. Individuals residing in urban setting were more than three times more likely to have good knowledge about stroke; and it is in line with a study conducted in Ugandan population [16], which might be justified as urban residents are exposed to new information; most of them are social media followers, and because of the increasing prevalence of chronic disease they are interested to know the complication of hypertension, they have health facilities around them, and they have opportunity for education.

## 5. Limitation

Since the study was institutional based and excludes hospitalized patients, generalization of the findings to the general populace is limited; in addition, since it was confined to public hospital, hypertensive patients who are considered economically sufficient and educated may have follow-up at another private hospital and the study did not assess them. Moreover, the study is limited by the fact that it was cross-sectional and used close-ended questions. This might have limited the participants' responses regarding their knowledge and attitudes towards stroke. However, the answer choices for each question were worded to cover a wide range of response possibilities.

## 6. Conclusion

The present study showed that knowledge regarding stroke risk factors and warning signs was significantly low among hypertensive patients, in which majority of participants were unable to identify any risk factors and warning signs of stroke. Being young, urban residence, being educated, and having sufficient income were significant predictors of good knowledge towards stroke risk factors and warning signs. These findings suggest the need for all stakeholders to emphasize stroke education to help individuals to understand and manage stroke risk factors as well as its warning signs through public or social media and school and health education crucially for low-income high risk subjects.

## Figures and Tables

**Table 1:** Sociodemographic and clinical characteristics of hypertensive patients at Felege Hiwot Referral Hospital in Bahir Dar Town, Ethiopia, 2018.

Variables	Frequency	Percent (%)
Age category		
** **<45	67	24.1%
** **≥ 45	211	75.9%

Sex		
Female	145	52.2%
Male	133	47.8%

Residence		
Rural	137	49.3%
Urban	141	50.7%

Religion		
** **Orthodox	220	79.1%
** **Muslim	38	13.7%
** **Protestant	17	6.1%
** **Adventist	2	.7%
Catholic	1	.4%

Marital status		
Single	16	5.8%
Married	200	71.9%
Divorced	22	7.9%
Widowed	40	14.4%

Monthly income		
Sufficient	103	37.0%
Insufficient	175	63.0%

Level of education		
Unable to read	154	55.4%
Educated	124	45.6%

Occupation		
Government worker	62	23.3%
Merchant	41	14.7%
Housewife	34	12.2%
Student	13	4.7%
Labour worker	17	6.1%
Farmer	82	29.5%
Others	29	10.4%

BMI		
Normal	253	91.0%
Overweight	22	7.9%
Obese	3	1.1%

Smoking		
Yes	8	2.9%
No	270	97.1%

Alcoholism		
Yes	22	7.9%
No	256	92.1%

Chat chewing		
Yes	1	0.4%
No	277	99.6%

**Table 2:** Socio-demographic characteristics associated with general knowledge of hypertensive patients.

Variables	Knowledge		COR (95%CI)	AOR(95%CI)	P-value
	Good	poor			
Age					
<45	45	182	3.068(1.613-5.837)	2.59(1.225-6.20)	*0.001∗*
≥45	22	29	1.00	1.00	

Sex					
Female	18	127	1.00		
Male	33	100	2.328(1.238—4.377)		

Marital status					
Single	7	9	3.033(3.30-27)		
Married	41	59	10.05(1.33-7.53)		
Divorced	2	22	3.93(0.33-4.5)		
Widowed	1	39	1.00		

Residence					
Rural	4	134	1		
Urban	44	95	12.41(4.745-32.470)	3.549(1.191-10.573)	*0.023∗*

Level of education					
Unable to read	5	149	1.00	1.00	
Able to read and write	46	78	17.57(6.711-46.024)	7.12(2.298-22.10)	*0.001∗*

Income					
Sufficient	33	70	5.731(2.943-11.161)	2.756(1.225-6.20)	*0.014∗*
Insufficient	16	159	1.00	1.00	

*NB*: variables having a (P ≤ 0.2) in bi variable (unadjusted) analysis included in the multivariable (adjusted) analysis. *∗*Statistically significant at p-value ≤ 0.05. COR = Crude Odd Ratio. AOR = Adjusted Odd Ratio.

## Data Availability

The datasets used and analysed during the study are available from the corresponding author on reasonable request.

## Abbreviations

AOR: Adjusted Odds Ratio
CI: Confidence Interval
COR: Crude Odds Ratio
Epi info: Statistical Package for Epidemiological Information Analysis
SPSS: Statistical Package for Social Sciences.
